# Cerium Oxide Nanoparticles Absorption through Intact and Damaged Human Skin

**DOI:** 10.3390/molecules24203759

**Published:** 2019-10-18

**Authors:** Marcella Mauro, Matteo Crosera, Matteo Monai, Tiziano Montini, Paolo Fornasiero, Massimo Bovenzi, Gianpiero Adami, Gianluca Turco, Francesca Larese Filon

**Affiliations:** 1 Clinical Unit of Occupational Medicine, Department of Medical Sciences, University of Trieste, Via della Pietà 19, 34100 Trieste, Italy; bovenzi@units.it (M.B.); larese@units.it (F.L.F.); 2 Department of Chemical and Pharmaceutical Sciences, University of Trieste, Via Giorgieri 1, 34127 Trieste, Italy; mcrosera@units.it (M.C.); matteo.monai@phd.units.it (M.M.); tmontini@units.it (T.M.); pfornasiero@units.it (P.F.); gadami@units.it (G.A.); 3 ICCOM-CNR Trieste research unit and INSTM Trieste research unit, Via Giorgeri 1, 34127 Trieste, Italy; 4 Department of Medical Sciences, University of Trieste, Piazza dell’Ospitale 1, 34125 Trieste, Italy

**Keywords:** Franz cells, in vitro, cerium oxide nanoparticles, transdermal absorption

## Abstract

Cerium oxide (CeO_2_) nanoparticles (NPs) are used in polishing products and absorbents, as promoters in wound healing, and as organopesticide decontaminants. While systemic bioaccumulation and organ toxicity has been described after inhalation, data on CeO_2_ NPs’ transdermal permeation are lacking. Our study was an in vitro investigation of the permeation of 17-nm CeO_2_ NPs dispersed in synthetic sweat (1 g L^−1^) using excised human skin on Franz cells. Experiments were performed using intact and needle-abraded skin, separately. The average amount of Ce into intact and damaged skin samples was 3.64 ± 0.15 and 7.07 ± 0.78 µg cm^−2^, respectively (mean ± SD, *p* = 0.04). Ce concentration in the receiving solution was 2.0 ± 0.4 and 3.3 ± 0.7 ng cm^−2^ after 24 h (*p* = 0.008). The Ce content was higher in dermal layers of damaged skin compared to intact skin (2.93 ± 0.71 µg cm^−2^ and 0.39 ± 0.16 µg cm^−2^, respectively; *p* = 0.004). Our data showed a very low dermal absorption and transdermal permeation of cerium, providing a first indication of Ce skin uptake due to contact with CeO_2_.

## 1. Introduction

In the last decade, nanotechnology has found applications in industrial processes, consumer products, and biomedicine due to the unique properties of nano-sized materials. At the nanometric scale, particles not only have a great surface area to mass ratio, but also expose a higher fraction of surface undercoordinated sites (e.g., kinks, step sites) and may have different electronic properties with respect to their bulky counterparts due to quantum confinement effects. This translates into different chemical and physical properties, which can be exploited (e.g., catalysis, sensors, and electronics) [1]. 

On the other hand, the particular technological advancement supported by these nanotechnologies has not been accompanied by exhaustive toxicological studies designed to assess any unforeseen health effects that nanomaterials may have on final consumers and workers.

Most of the data available to date with regard to nanoparticles (NPs) exposure risk assessment has been focused on the inhalation pathway, which represents the main route involved in xenobiotic systemic uptake when particles are dispersed in air. Nevertheless, there are other routes which deserve to be investigated, such as skin uptake, since this could represent an unrecognized systemic uptake pathway when nanoparticles are handled in occupational scenarios or when intentionally applied on the skin (e.g., in consumer goods) [2]. 

Cerium oxide nanoparticles (CeO_2_ NPs) have several applications, such as high surface area support in oxidation and hydrogenation catalysts and as an additive in fuels to reduce particulate emissions [3,4,5]. Notably, it has been estimated that large amounts of these NPs will be released in the environment as a consequence of this proposed use, despite their health effects not being completely known and potentially dangerous [6,7]. Clar et al. demonstrated an environmental release and a dermal transfer of CeO_2_ NPs used as UV inhibitors on outdoor surfaces [8].

Occupational exposure is also possible for people working with polishing materials for electronic screens and optic glass [9,10], as well as those working with absorbent compounds in systems to clean heavy metals from aqueous systems [11].

Moreover, based on their excellent catalytic and antioxidant activity in many biological contexts, CeO_2_ NPs have been proposed as pharmacological treatments for diseases associated with oxidative stress, such as neurodegenerative pathologies, autoimmune diseases, diabetes, cancers and wound repair [12,13,14,15,16]. Finally, CeO_2_ NPs demonstrate a promising activity in skin decontamination of organophosphorus compounds [17].

Despite the commercial interest of these NPs, their health effects are not completely known, and studies conducted on this topic show conflicting results. While some authors found toxic effects, others highlight CeO_2_ antioxidant properties. In the first case, irritant effects and airflow obstruction have been demonstrated in lungs after CeO_2_ NPs inhalation, while cyto-, geno-, hepato- and neurotoxicity have been observed but not well characterized [18,19]. On the other hand, CeO_2_ NPs have been shown to act as free-radical scavengers and have been proposed as promoter agents in wound healing [20,21,22].

For all the aforementioned, the European Agency for Safety and Health at Work [23] has listed CeO_2_ NPs in the top five nanoparticles worthy of investigation as a priority.

Since some applications of CeO_2_ NPs (e.g., promoters in wound healing and decontaminants in pesticide dermal fouling) presuppose direct contact with the skin, their use has to be evaluated from a safety point of view, since a systemic uptake may pose toxicological side effects.

The aim of the present study was to assess CeO_2_ NPs transdermal permeation using intact and damaged excised human skin samples in order to evaluate their dermal exposure safety profile.

## 2. Results

### 2.1. Nanoparticles Characterization

**Figure 1:**
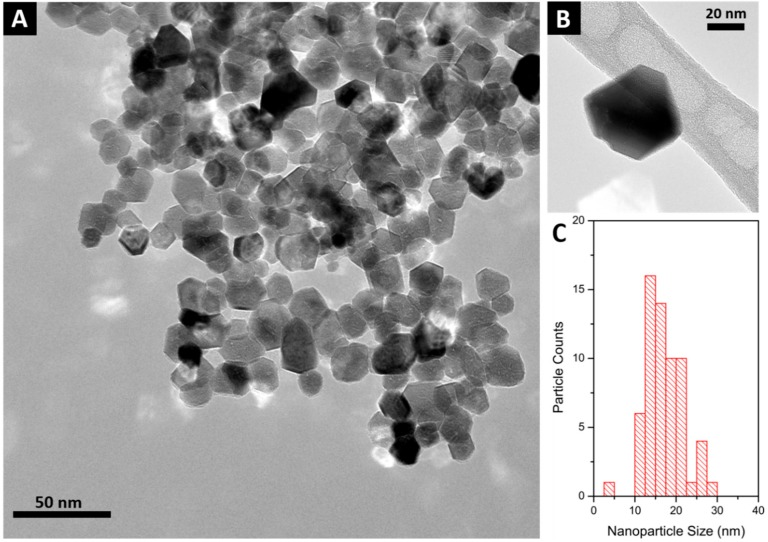
(**A**) Representative TEM image of CeO_2_ nanoparticles (NPs) synthesized in synthetic sweat at 120 °C for three days. (**B**) Close-up of a polyhedral NP. (**C**) CeO_2_ NPs size distribution.

The concentration of 15 mM of the starting NPs dispersion was confirmed by the ICP-OES analysis. Representative TEM images of the CeO_2_ nanoparticles used in this study are reported in Figure 1. The NPs had a rather large size distribution of 17 ± 5 nm, and presented various shapes, ranging from spherical to polyhedral.

**Figure 2:**
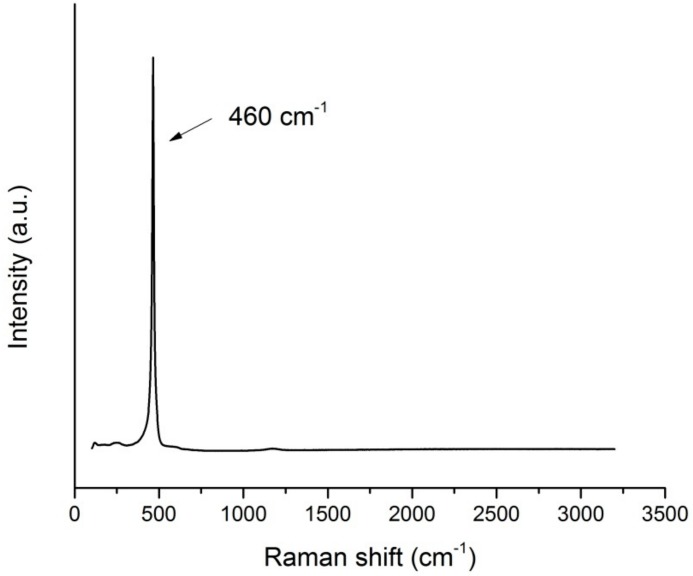
Raman spectrum of CeO_2_ nanoparticles synthesized in synthetic sweat.

The formation of crystalline CeO_2_ was confirmed by Raman spectroscopy (Figure 2), with the presence of the characteristic peak at 464 cm^−1^, plus two broad and barely visible peaks at lower (~255 cm^−1^) and higher (~590 cm^−1^) energy [24]. The sharp and symmetric shape of the peak, and its Raman shift (464 cm^−1^), qualitatively indicated a large size of the ceria particles.

The cerium concentration in the donor phase, which was collected at the beginning of the test after the removal of the NPs by ultrafiltration, was less than 0.05% of the starting suspensions and did not change at the end of the experiments.

### 2.2. Franz Diffusion Cells Experiments

In the experimental condition, the used Ce did not permeate the intact skin since the concentration in the receiving phase was the same as that of the blank cells (2.0 ± 0.4 ng cm^−2^). However, it reached 3.3 ± 0.7 ng cm^−2^ for damaged skin (*p* = 0.008).

**Figure 3:**
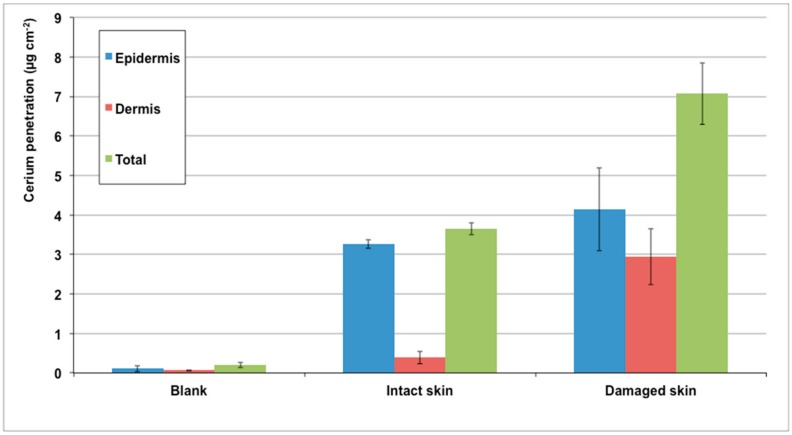
Cerium penetration (µg cm^−2^) into the skin layers (epidermis, dermis, and total skin) after 24 h of exposure to a dispersion of CeO_2_ NPs in synthetic sweat.

The amount of Ce (µg cm^−2^) inside whole skin was 3.64 ± 0.15 for intact skin, 7.07 ± 0.78 for damaged skin, and 0.19 ± 0.06 for blank cells (mean ± SD). Figure 3 shows the Ce amount in each layer of intact and damaged skin.

**Figure 4:**
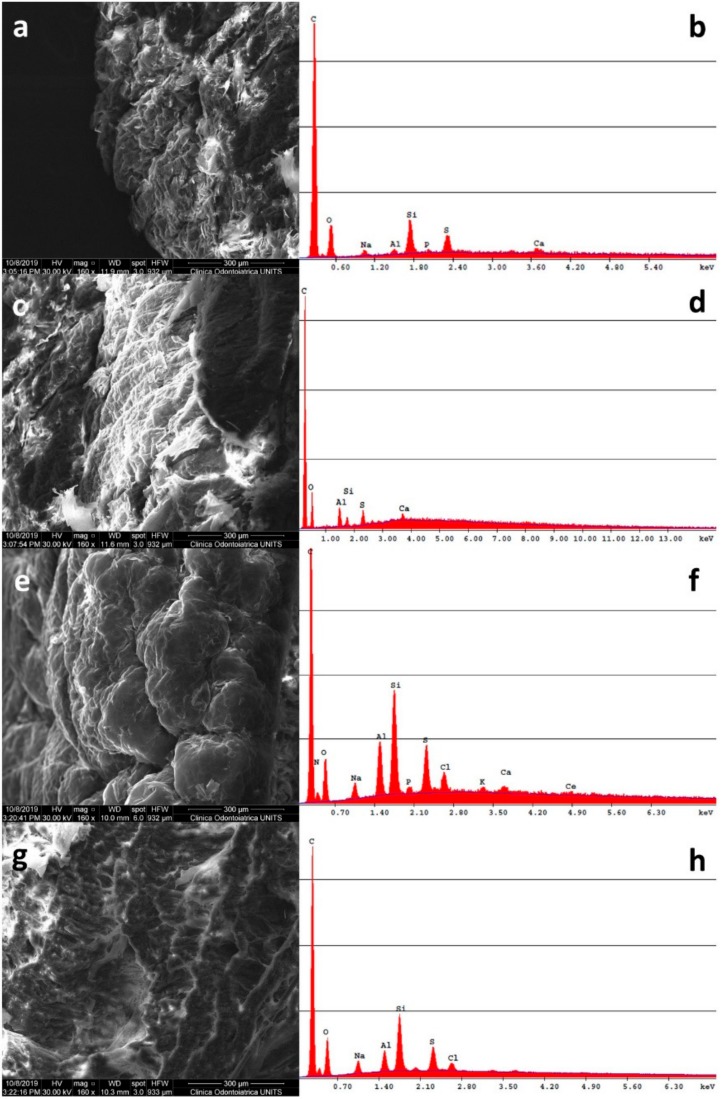
Representative SEM-EDS micrographs of skin samples treated with CeO_2_ NPs and EDS spectra in full-frame acquisition mode: (**a**) epidermis of a blank cell and (**b**) relative EDS spectrum; (**c**) dermis of a blank cell and (**d**) relative EDS spectrum; (**e**) epidermis of an intact skin sample and (**f**) relative EDS spectrum; (**g**) dermis of an intact skin sample and (**h**) relative EDS spectrum. Bar = 300 µm for (**a**,**c**,**e**,**g**).

**Figure 5:**
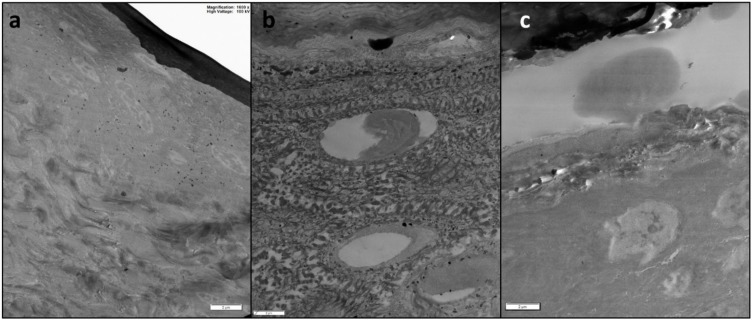
Representative TEM micrographs of skin samples treated with CeO_2_ NPs: (**a**) blank cell; (**b**) intact skin; and (**c**) damaged skin. Bar = 2 µm.

Microscopic analysis confirmed the low penetration and permeation of CeO_2_ NPs since only a small signal of Ce was revealed in the epidermis of an intact skin sample (Figure 4) but NPs were not visualized by TEM investigation (Figure 5).

## 3. Materials and Methods

### 3.1. Chemicals

All chemicals were of analytical grade. Urea, sodium chloride, sodium hydrogen phosphate, and potassium dihydrogen phosphate were purchased from Carlo Erba (Milan, Italy). Lactic acid (90% *w*/*w*) was bought from Acros Organics (Geel, Belgium). Nitric acid (69% *w*/*w*), ammonium hydroxide (25% *w*/*w*), boric acid, cerium ammonium nitrate (CAN, 99.99%), and hydrofluoric acid (48% *w*/*w*) were from Sigma Aldrich (Milan, Italy).

Reagent-grade water (Milli-Q) was produced with a Millipore purification pack system.

### 3.2. Nanoparticles Synthesis and Characterization

The CeO_2_ NPs investigated in this study were synthesized by a hydrothermal route using CAN and a synthetic sweat solution as precursors. The synthetic sweat solution used was the same as the one employed as donor fluid, consisting of 0.5% sodium chloride, 0.1% urea, and 0.1% lactic acid in Milli-Q water, and pH was adjusted to 4.5 using a concentrated ammonia solution. Typically, 165 mg of CAN was dissolved in 20 mL of synthetic sweat to obtain a final concentration of 15 mM, and the solution was introduced into a 25 mL autoclave fitted with a Teflon liner. The autoclave was then placed in a static oven pre-heated to 120 °C for 3 days.

The final dispersion was filtered over Amicon filters and the Ce concentration remaining in the supernatant solution was determined by inductively coupled plasma optical emission spectrometry (ICP-OES).

The resulting powder was washed three times with Milli-Q water and dried at 120 °C overnight before re-dispersion into synthetic sweat for skin permeation tests.

Transmission electron microscopy (TEM) was performed with an EM208 (Philips, Heidhoven, The Netherlands) with an SIS Morada high-definition acquisition system and an iTEM digital image acquisition system (FEI Italia, Milan, Italy). Before observation, a droplet of CeO_2_ dispersed in synthetic sweat was deposed on carbon grids. The carbon grids were then left to dry for one day. The size distribution of CeO_2_ was determined for 70 particles using TEM images.

Raman spectra were recorded on an inVia Renishaw microspectrometer equipped with an Nd:YAG laser using an excitation wavelength of 532 nm.

At the time of the experiments, CeO_2_ had been removed from aqueous solutions in three different aliquots (2 mL) of the freshly prepared solution by means of ultrafiltration in centrifuge at 6000 rpm for 30 min using the Amicon Ultra-4 centrifugal filters (10K MWCO). This was done in order to evaluate the percentage of ionized metal in the donor phase. The filtered solutions were collected and analyzed to determine cerium concentrations by means of ICP-OES.

The ionization of the donor phase was checked after 24 h exposure, and the ultrafiltration procedure was repeated on the donor phases once they had been removed from the cells at the end of the experiments.

### 3.3. Preparation of Skin Membranes

Human abdominal full-thickness skin was obtained as surgical waste from two female donors aged 45–65 years, who underwent plastic surgery for esthetic reasons. Ethical committee approval was obtained.

After the skin excision, subcutaneous fat was removed with a scalpel blade and hair was shaved from the epidermis. Skin samples were stored at −25 °C for a period up to, but not exceeding, four months. It has been demonstrated that this procedure does not damage skin barrier properties [25,26]. On the day of the experiment, skin samples were defrosted in a physiological solution at room temperature for a 30-min period and 2 × 2 cm^2^ pieces were cut from each skin specimen and mounted separately on the diffusion cells.

Skin integrity was assessed using the transepidermal water loss (TEWL) method as described by Guth et al. [27]. Cells with a value of >10 g m^−2^ h^−1^ were considered to be damaged and rejected. Donors were men and women with an age range of 45–71 years. The study was approved by the Trieste Hospital Ethical Committee n° 236/2007.

### 3.4. In Vitro Diffusion System

Percutaneous absorption studies were performed using static diffusion cells following the Franz method [27], OECD test guideline 428 [28], and protocols suggested by EDETOX [29]. The receptor compartment had a mean volume of 4.5 mL and was maintained at 32 °C (i.e., the physiological average temperature of a human hand) by means of circulating thermostated water in the jacket surrounding the cell. The physiological solution used as the receptor phase was prepared by dissolving 2.38 g of Na_2_HPO_4_, 0.19 g of KH_2_PO_4_, and 9 g of NaCl into 1 L of Milli-Q water (final pH 7.35). The salt concentration in the receiving fluid was approximately the same as that found in blood. The physiological solution used as the receiving phase was continuously stirred using a Teflon-coated magnetic stirrer. Each piece of skin was clamped between the donor and the receptor compartment. Skin samples from donor 1 and donor 2 were equally divided between experiments one and two. The mean exposed skin area was 0.95 cm^2^ and the average membrane thickness was 1 mm. Two different experiments were conducted using intact and damaged skin.

In intact skin permeation experiments at time 0, the exposure chambers of three Franz diffusion cells were filled with 0.220 mL of the donor solution, corresponding to an amount of CeO_2_ of 0.6 mg cm^−2^, to ensure an infinite dose. The applied dose was the same as previous studies on nanoparticle permeation in order to better compare the results of the experiments [30,31].

After 24 h, the donor phase of each diffusion cell was removed and recovered. After the integrity test, the receiving solutions and the skin pieces were also removed and stored in the freezer for quantitative analyses.

The total cerium concentrations in the donor phases were confirmed after the experiments by means of ICP-OES.

Skin permeation experiments on abraded skin were conducted following the same procedure. The skin was prepared following the protocol reported by Bronaugh and Steward [32], where skin was abraded by drawing the point of a 19-gauge hypodermic needle across the surface (six marks in one direction and six marks made perpendicular).

For each experiment, one cell was added as a blank. The blank cells were treated in the same way as the other cells, with the exception that only synthetic sweat without NPs was used in the donor compartment.

Each experiment was repeated two times in order to use the skin of four different donors. As equipment use was static, there was no relationship between the cells tested. Therefore, each represented an independent evaluation, for a total of six cells with intact skin, six cells with damaged skin, and four blank cells.

### 3.5. Skin Digestion after the Experiment

After the experiment, the skin pieces were washed three times with Milli-Q water to remove residual CeO_2_ NPs from the skin surface. They were then removed from the diffusion cells and separated into epidermis and dermis by heat shock, by being immersed in water at 60 °C for 1 min before freezing at −25 °C. At the time of the analysis, the skin membranes were dried for 2 h at room temperature, weighed, and acid-digested in a closed microwave system (Multiwave PRO, Anton Paar) using a mixture of 3 mL of HNO_3_ (69%) and 0.5 mL of HF (48%). The mineralization was performed through two heating steps. In the second step, H_3_BO_3_ was added in order to buffer the excess HF. The solutions obtained from the mineralization process were diluted up to a volume of 10 mL by adding Milli-Q water before ICP-OES analysis.

### 3.6. Analytical Measurements

ICP-MS Nexion 350X with an ESI autosampler (Perkin Elmer, USA instrument, Perkin Elmer, Waltham, Massachusetts, MA, USA) was used to determine total cerium concentration in the receiver phases. A six-point standard curve obtained by the dilution of cerium standard solution for ICP-MS analysis (by Sigma Aldrich, Milan) was used for ICP-MS measurements (0.01–10 μg L^−1^, ion mass 140 u.m.a.). The limit of detection of cerium was 0.001 μg L^−1^ for ICP-MS and the precision of the measurements as repeatability (relative standard deviation (RSD) %) for the analysis was <5%.

Total cerium concentration in donor phases and in the solutions resulting from the mineralization of the skin samples were performed by (ICP-OES) using an Optima 8000 Spectrometer (PerkinElmer) equipped with an S10 Autosampler. ICP-OES analyses were conducted using a calibration curve obtained by dilution (range: 0–10 mg L^−1^) of cerium standard solution (by Sigma Aldrich, Milan, Italy). The limit of detection (LOD) at the operative wavelength of 413.764 nm was 0.01 mg L^−1^. The precision of the measurements expressed as RSD % for the analysis was always less than 5%.

### 3.7. Skin Fixation Protocol and Microscopic Analysis

#### 3.7.1. Transmission Electron Microscopy (TEM)

After removal, small sections were taken from selected skin samples (one for each type of exposure), fixed in a solution of glutaraldehyde in 0.1 M cacodylate buffer (pH 7.3), and post fixed with 1% osmium tetroxide for 1 h at 4 °C. Post-fixed samples were dehydrated with an ascending ethanol series ending with 100% ethanol and then embedded in Down epoxy resin. Semi-fine and ultra-thin sections were prepared with an ultra-microtome, where ultra-thin sections were double stained with lead citrate and uranyl acetate and observed with a transmission electron microscope (EM208; Philips, Heidhoven, The Netherlands). More details in [33].

#### 3.7.2. Scanning Electron Microscopy and X-ray Microanalysis (SEM-EDS)

From the same selected skin samples, small sections were fixed in paraformaldehyde (4%) for 3 h and then mounted on aluminum stubs and subsequently carbon coated (Sputter Coater K550X, Emitech, Quorum Technologies Ltd., Laughton, UK). Scanning electron microscopy (Quanta250 SEM, FEI, Hillsboro, OR, USA) was performed in secondary electron detection mode. The working distance was adjusted in order to obtain suitable magnification, and the accelerating voltage was 30 kV. The elemental composition was determined using an EDS probe (Quanta250 FEI with EDAX probe, Hillsboro, OR, USA) working in full-frame acquisition.

### 3.8. Statistical Analysis

Cerium concentration data (μg cm^−3^) in the receptor solution were converted to the Ce amount that penetrated per skin surface unit (μg cm^−2^). Data analysis was performed with Excel for Windows, release 2007, and Stata Software, version 11.0 (StataCorp LP, College Station, TX, USA). Skin absorption data were reported as mean ± standard deviation (SD). The difference among independent data was assessed by means of the Mann–Whitney test. A *p*-value < 0.05 was considered significant.

## 4. Discussion and Conclusions

CeO_2_ NPs are widely used in many technological and biomedical applications, due mainly to their absorbent and reactive oxygen species (ROS) scavenger properties, resulting in general population and specific working group exposure. Despite this, their possible systemic uptake after dermal exposure has not yet been investigated. The only toxicological studies conducted in vivo demonstrated cytoxicity, genotoxicity, hepatoxicity, and neurotoxicity after bioaccumulation following inhalation [4,15].

Therefore, their proposed dermal use as promoters in wound healing and as decontaminants in pesticide dermal fouling has to be evaluated from a safety standpoint, since a systemic uptake may pose toxicological side effects.

Our study found, for the first time, that CeO_2_ NPs could not permeate intact skin, but this permeation was possible—if at a low level—using an abraded skin protocol. A low amount of cerium was present in intact and damaged skin samples after prolonged exposure (24 h) to CeO_2_NPs. Cerium concentration inside damaged skin was nearly double compared to intact skin (*p* = 0.004, Mann–Whitney test) and significantly higher compared to blank cells. These results are comparable to those of previous studies on the cutaneous absorption of other metal oxide nanoparticles [30,31]. This behavior is probably due to the very low ionization of these metal oxides in synthetic sweat, resulting in a small concentration of free metal ions in the donor phase being able to cross over the physiological barriers. The NPs’ capability of permeating the dermal layers is lower with respect to the free ions, and depends on their physicochemical characteristics [2].

These data represent an encouraging result for CeO_2_ NPs applications that involve direct skin exposure.
